# Supplementary material for: Brassinosteroids Enhance Low-Temperature Resistance by Promoting the Formation of Sugars in Maize Mesocotyls
Source: Plants (Basel). 2025 Aug 22;14(17):2612. doi: 10.3390/plants14172612 (PMC12430796; doi:10.3390/plants14172612)
Supplement: Supplementary file 1 [file plants-14-02612-s001.zip › plants-3789682-supplementary.pdf]

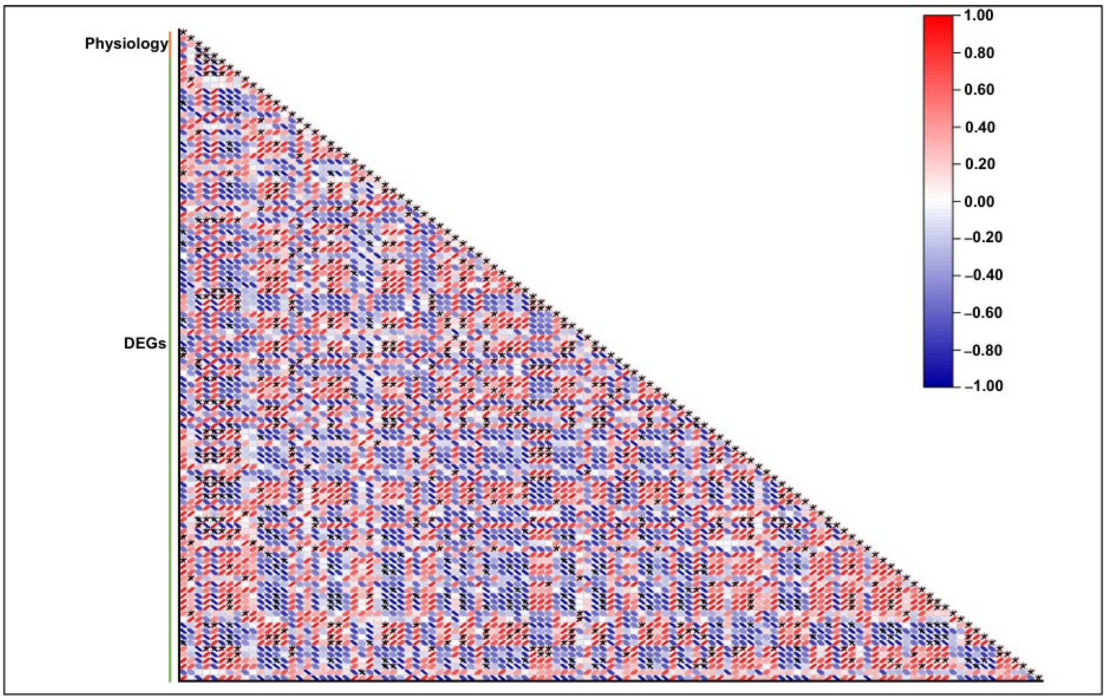

Figure S1. Correlation analysis of the physiological contents of glucose, sucrose and starch with differentially expressed genes (DEGs) in the starch and sucrose metabolism pathway.

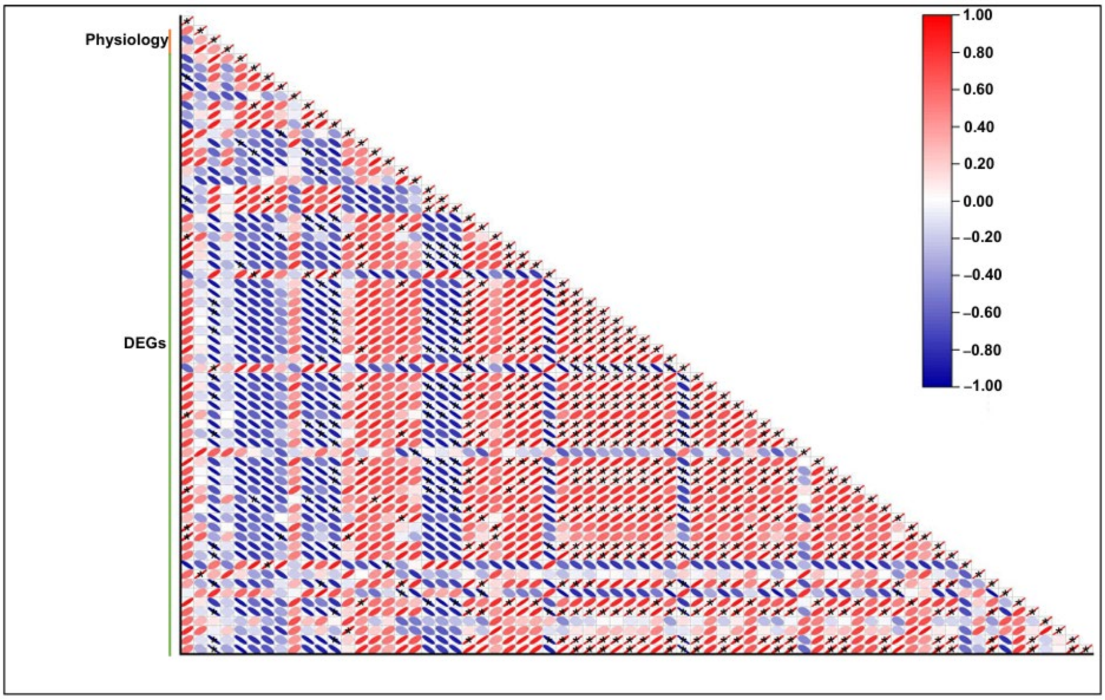

Figure S2. Correlation analysis of the physiological contents of glucose, sucrose and starch with differentially expressed genes (DEGs) in the Glycolysis/Gluconeogenesis pathway.
